# Cigarette Smoke-Induced Pulmonary Inflammation and Autophagy Are Attenuated in Ephx2-Deficient Mice

**DOI:** 10.1007/s10753-016-0495-z

**Published:** 2016-12-27

**Authors:** Yunxiao Li, Ganggang Yu, Shaopeng Yuan, Chunting Tan, Puqiao Lian, Lixia Fu, Qi Hou, Bo Xu, Haoyan Wang

**Affiliations:** 1 The Department of Respiratory Medicine, Beijing Friendship Hospital, Capital Medical University, No. 95 Yong An Road, Xichen District, Beijing, 100050 China; 2 Beijing Key Laboratory of New Drug Mechanisms and Pharmacological Evaluation Study, Institute of Materia Medica, Chinese Academy of Medical Sciences and Peking Union Medical College, Beijing, 100050 China

**Keywords:** Ephx2, epoxyeicosatrienoic acids, inflammation, autophagy, cigarette smoke

## Abstract

Cigarette smoke (CS) increases the risk of chronic obstructive pulmonary disease (COPD) by causing inflammation, emphysema, and reduced lung function. Additionally, CS can induce autophagy which contributes to COPD. Arachidonic acid-derived epoxyeicosatrienoic acids (EETs) have promising anti-inflammatory properties that may protect the heart and liver by regulating autophagy. For this reason, the effect of decreased soluble epoxide hydrolase (sEH, Ephx2)-mediated EET hydrolysis on inflammation, emphysema, lung function, and autophagy was here studied in CS-induced COPD *in vivo*. Adult male wild-type (WT) C57BL/6J and Ephx2^−/−^ mice were exposed to air or CS for 12 weeks, and lung inflammatory responses, air space enlargement (emphysema), lung function, and autophagy were assessed. Lungs of Ephx2^−/−^ mice had a less pronounced inflammatory response and less autophagy with mild distal airspace enlargement accompanied by restored lung function and steady weight gain. These findings support the idea that Ephx2 may hold promise as a therapeutic target for COPD induced by CS, and it may be protective property by inhibiting autophagy.

## INTRODUCTION

Chronic obstructive pulmonary disease (COPD) is an inflammatory disease characterized by emphysema, persistent airflow limitations, and reduced lung function [31]. It is the third leading cause of death worldwide and it represents an important public health challenge [44]. Cigarette smoking (CS) is the main risk factor for developing COPD and airway inflammation, which contributes to airway remodeling and pulmonary emphysema [42].

Chronic exposure to CS leads to lung inflammation and increased levels of macrophages [37], neutrophils [8, 16], and dendritic cells (DCs) [40]. An uncontrolled and prolonged inflammatory response may destroy lung tissue and promote remodeling [5, 36]. Elimination of inflammation is a key treatment target for COPD.

Epoxyeicosatrienoic acids (EETs), arachidonic acid-derived epoxides, are important regulators of inflammation and vascular homeostasis [6]. Manipulating them may be a suitably anti-inflammatory pharmacological strategy. Notably, arachidonic acid is metabolized by CYP epoxygenase enzymes, CYP2C and CYP2J subfamilies, to biologically active EETs [46]. Then EETs are hydrolyzed by soluble epoxide hydrolase (sEH, Ephx2) to dihydroxyeicosatrienoic acid (DHETs), which have less biological function [46]. EETs are found in numerous organs such as the lung, kidney, heart, liver, and brain [12, 47]. They have anti-inflammatory effects on human bronchi [23]. A sEH inhibitor has been found to attenuate CS-induced inflammation in mouse models as well [32]. Nording and colleagues showed TNF-α mRNA expression to be significantly decreased in animals treated with a sEH inhibitor and in Ephx2^−/−^ animals in response to sub-chronic tobacco smoke (TS) exposure [30]. Previous studies have suggested that EETs disrupt NF-κB signaling to exert anti-inflammatory effects [9, 29]. However, the phenotypic influence of Ephx2 disruption on inflammation in response to chronic CS is unclear.

Autophagy is a dynamic process involving cytoplasmic organelle and protein turnover via a lysosome-associated degradation pathway. Material is engulfed by autophagosomes and then fuses with lysosomes to be degraded by lysosomal hydrolases. Starvation, oxidative stress, cytokines, and xenobiotics can induce autophagy, suggesting it is important for maintaining cellular homeostasis and adapting to adverse environments [13, 17, 18]. Autophagy contributes to lung disease, exerting effects on fundamental cellular processes such as inflammation, apoptosis, redox balance, and proliferation [21, 26, 27]. COPD patients have more autophagy in epithelial cells than in healthy individuals [25–27] and previous studies have suggested that autophagy contributes to inflammation and emphysema in COPD [1, 3, 4]. López-Vicario and Samokhvalov reported that EETs modulate autophagic response and inhibit inflammation in fatty livers of obese subjects and improved starved cardiac cell viability and recovery [19, 35]. However, the effect of EETs or decreased soluble epoxide hydrolase (sEH, Ephx2)-mediated EET hydrolysis on autophagy in CS-induced COPD is less well understood. For this reason, we here investigated whether Ephx2 deficiency could modulate inflammation and autophagy in COPD and influences emphysema and lung function using Ephx2 (sEH) knockout (Ephx2−/−) and wild-type (WT) mice and a mouse model of CS-induced COPD.

## MATERIALS AND METHODS

### Mice

Ephx2^−/−^ mice were a gift from the Yi Zhu Laboratory (Tian Jin Medical University, Tian Jing, China). Mice were backcrossed with C57BL/6J mice for five generations prior to use to produce heterozygous Ephx2^+/−^ offspring. Ephx2^+/−^ offspring was intercrossed to generate Ephx2^+/+^ and Ephx2^−/−^ mice. All animals were housed in a temperature- and humidity-controlled room and kept on a 12:12-h light/dark cycle. Clean food and water were given *ad libitum*. Tail snips were obtained for DNA extraction to identify murine genotype with PCR. Primers were as follows: F1: 5′-TGG CAC GAC CCT AAT CTT AGG TTC-3′; R1: 5′-TGC ACG CTG GCA TTT TAA CAC CAG-3′; R2: 5′-CCA ATG ACA AGA CGC TGG GCG-3′ [39]. Primer F1/R1 predicts a 338-base pair (bp) amplicon and indicates the Ephx2^+/+^, primer F1/R2 predicts a 295-bp product and it indicates Ephx2^−/−^ [39]. Primers were designed by TIANGEN Biotech (Beijing) Co., Ltd and genotypic analysis was examined on 3% agarose gel (Lonza Rockland, Inc., Rockland, ME, USA). All animal studies were approved by the Animal Care and Use Committee of Capital Medical University.

### Animal CS Exposure

WT and Ephx2^−/−^ mice (10 to 12 weeks old, ∼18–20 g) were randomly divided into two groups (*N* = 10). WT controls and KO controls were exposed to filtered air. WT-CS and KO-CS groups were whole-body exposed to CS from ten cigarettes (11 mg tar, 0.8 mg nicotine/cigarette; Daqianmen, China) in a sealed 50-L metal exposure chamber. Mice were exposed to 10 cigarettes for 60 min per exposure, once daily for 12 weeks. During exposure, smoke was maintained between 80 and 100 ppm at 18% oxygen. Animal weight was measured once every 2 weeks.

### Preparation of BALF and Cell Counts

After 12 weeks CS exposure, mice were sacrificed and bronchoalveolar lavage fluid (BALF) was obtained by cannulating the trachea and lavaging it three times with 0.5 ml of 0.9% sodium chloride. BALF cells were centrifuged with PBS at 1000×*g* for 10 min at 4 °C. Supernatants were collected and stored at −80 °C to measure inflammatory factors. Pelleted BALF cells were resuspended in 1 ml PBS, and erythrocytes, neutrophils, and monocytes were quantified with a LH 750 (Beckman Coulter, Miami, FL, USA).

### Cytokine Analysis

TNF-α and IL-1β in BALF were measured with ELISA using mouse TNF-α and IL-1β ELISA MAX™ Deluxe (BioLegend, San Diego, CA, USA) according to manufacturer’s instructions. Each sample was assayed in triplicate.

### Lung Histology and Quantification of Emphysema

After mice were sacrificed, their tracheas and lungs were removed and inflated with 4% paraformaldehyde at a pressure of 25 cm H_2_O. Tissues were embedded in paraffin, and 5-μm-thick sections were stained with hematoxylin and eosin (H&E). Alveolar size was quantified using the mean linear intercept (Lm) of the airspace which is a measurement of airspace enlargement (emphysema). Lm was measured in 10 randomly selected fields of tissue sections observed at a magnification of ×200.

### Lung Function

Lung function was assessed with a ventilator for small animals (flexiVent™; SCIREQ, Montreal, QC, Canada). After 12 weeks of daily exposure to air or CS, mice were anesthetized with thiopental (250 mg/kg, ip, Sigma-Aldrich, St. Louis, MO, USA) and tracheostomized. Then, a 20G angiocatheter (BD Insyte, Sandy, UT, USA) was inserted into the trachea and a catheter was used to connect the angiocatheter and the ventilator. The tidal volume of the ventilator was set at 10 ml/kg, and respiratory rate was 120 breaths/min with a positive end-expiratory pressure of 3 cm H_2_O. Lung function was expressed as Newtonian resistance (Rn), respiratory system resistance (Rrs), respiratory system compliance (Crs), and respiratory system elasticity (Ers).

### Immunohistochemistry

Lungs were removed and fixed in 4% paraformaldehyde overnight and then embedded in paraffin to obtain three consecutive 5-μm thick sections. Sections were de-paraffinized in xylene and rehydrated through graded alcohol, followed by rinsing in PBS. Sections were placed in EDTA-antigen retrieval buffer and then exposed to 3% H_2_O_2_ for 10 min to block endogenous peroxidase activity at room temperature. Samples were blocked with sheep serum for 15 min and incubated in LC3B, Beclin 1, and NFκB primary antibodies (Cell Signaling, Beverly, MA, USA) diluted in blocking sera (1:200) at 4 °C overnight. After incubation, slides were washed three times and incubated with biotinylated secondary antibodies (Cell Signaling, Beverly, MA, USA). A hen enzyme reaction was performed using peroxidase substrate and sections were counterstained with hematoxylin and dehydrated through graded alcohol, mounted with neutral gum and observed under an inverted phase contrast microscope (LeicaDMI3000 B; Leica, Solms, Germany) at ×400 magnification. The immunostaining intensity assessment was performed using Image-Pro Plus 6.0 software. Mean integrated optical density (IOD) was used to express the antigen spot color intensity. The nuclear NF-κB staining was performed by counting staining nucleus. Each group contained three sections from different mice.

### Electron Microscopy

Lung tissues were fixed in 2.5% glutaraldehyde for 2 h and washed three times in PBS for 10 min at room temperature. Subsequently, tissues were post-fixed in 2% osmium tetroxide and washed three times in double-distilled H_2_O. Then tissue was dehydrated in a series of graded ethanol followed by embedding in agar 100 resin. Seventy nanometer sections were cut and post stained with uranyl acetate and lead citrate. Tissues were photographed with a FEI Morgagni 268D transmission electron microscope (FEI, Eindhoven, Netherlands) at 80 kV. Images were acquired using an 11 megapixel Morada charge-coupled device camera (Morada; Olympus). At least three different lungs per group at ×100,000 magnification were used to assess autophagosomes.

### Western Blot

Lung tissue was homogenized in ice-cold RIPA lysate buffer (Applygen Technologies Inc., Beijing, China) according to the manufacturer’s instructions. This was followed by centrifugation at 15,000×*g* for 15 min at 4 °C. Nuclear protein was extracted with extraction reagents (Applygen Technologies Inc.) and protein was quantified with the BCA method (Applygen Technologies Inc.). Samples (20 μg protein/lane) were separated using 10% SDS-PAGE and then transferred onto PVDF membranes. Membranes were blocked with 5% nonfat milk for 2 h at room temperature and then washed three times in TBST and incubated overnight with rabbit polyclonal antibody against Beclin 1, NFκB (1:1000 dilution, Cell Signaling Technology), β-actin (1:2000 dilution, Cell Signaling Technology), and Lamin B1 (Abcam, Cambridge, MA, USA) at 4 °C overnight. After three washes, membranes were incubated with goat antibody linked to horseradish peroxidase (Cell Signaling, Beverly, MA, USA) for 1 h, and antibody-antigen reactivity was measured with Western blot (Clinx Science Instruments Co., Ltd, China). Band density was quantified with ImageJ software.

## STATISTICS

Data were expressed as means ± SD. Statistical analysis was performed with SPSS 17.0 (SPSS Inc., Chicago, IL, USA). Differences between two groups were assessed with a Student’s *t* test. *Post hoc* pairwise multiple comparisons were performed using one-way ANOVA followed by a Bonferroni correction (significance was set at *P* < 0.05).

## RESULTS

### Ephx2^−/−^ Mice Have Fewer Weight Changes After CS Exposure

**Fig. 1:**
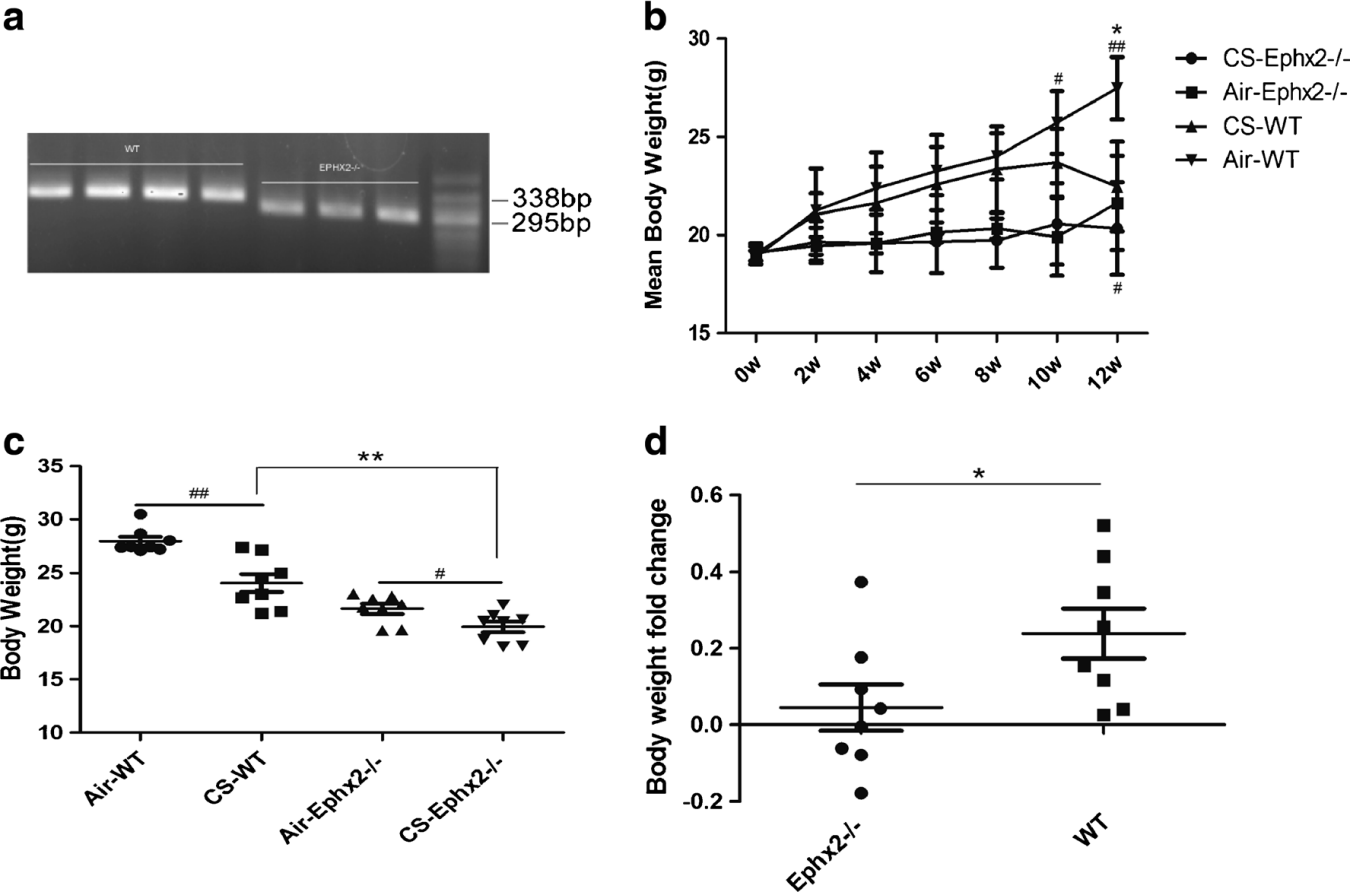
Weight of WT and KO mice changes after exposure to cigarette smoke. **a** Representative genotype analysis of WT and Ephx2^−/−^ mice. WT type mice produced a gene 338 bp in size and Ephx2^−/−^ mice produced a gene product 295 bp in size. **b**–**d** WT mice (*n* = 20) and Ephx2^−/−^ mice (*n* = 20) were exposed to air (air-WT, *n* = 10; air-Ephx2^−/−^, *n* = 10) or CS (CS-WT, *n* = 10; CS-Ephx2^−/−^, *n* = 10) for 12 weeks and weight was measured once every 2 weeks. **b** Weight gain over time. WT weight increased more than in the KO group. **c** After 12 weeks of treatment, mean weight of mice in the CS-WT group was lower than in the air-WT group and the mean weight of mice in the CS-Ephx2^−/−^ group was lower than that of mice in the air-Ephx2^−/−^group. **d** After 12 weeks of treatment, the Ephx2^−/−^ group weight fold-change was less than that of the WT group. Results are expressed as means ± SD. ^#^ *P* < 0.05, significant differences between CS-exposed group and its corresponding air-exposed group; ^##^ *P* < 0.01, significant differences between CS-exposed group and its corresponding air-exposed group; ^*^ *P* < 0.05, significant differences between CS-exposed WT mice and CS-exposed Ephx2^−/−^ mice, ^**^ *P* < 0.01, significant differences between CS-exposed WT mice and CS-exposed Ephx2^−/−^ mice.

WT and KO mouse weight was significantly reduced after 12 weeks of cigarette smoke exposure compared to those exposed to air (Fig. 1b). KO mice gained weight more slowly than the WT group in both exposure groups (Fig. 1c). After 12 weeks of cigarette smoke exposure, smoking KO animals weighed less than those in the WT group (Fig. 1b). However, after 12 weeks of treatment, fold-change difference between KO-Con and KO-CS groups was less than that between WT-Con and WT-CS groups (Fig. 1d). It may be that the stable weight observed in Ephx2^−/−^ mice is protective against cigarette smoke exposure.

### Ephx2^−/−^ Mice Have Less Airway Resistance in Response to CS Exposure

**Fig. 2:**
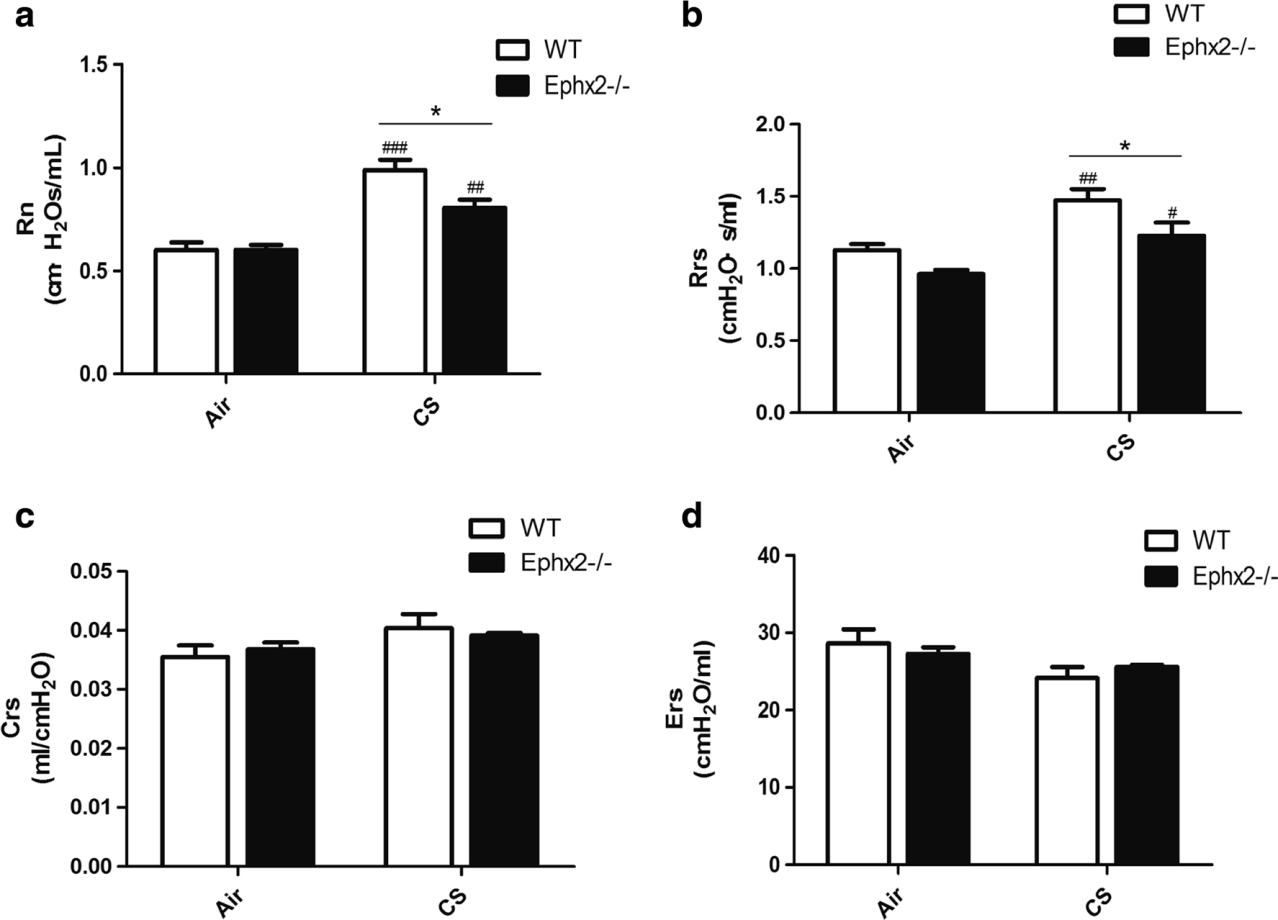
Ephx2^−/−^ mice have less airway resistance in response to CS exposure. **a** Rn; **b** Rrs; **c** Crs; and **d** Ers were measured in air- and CS-exposed WT and Ephx2^−/−^ mice, as described. **a**, **b** Rn and Rrs increased after 12 weeks of CS exposure in both groups. Rn and Rrs were significantly lower in CS-exposed Ephx2^−/−^ mice. **c**, **d** Crs and Ers did not differ between groups with CS exposure. Data are means ± SD (*n* = 3–4 mice/group). ^#^ *P* < 0.05, significant difference from corresponding air-exposed mice; ^##^ *P* < 0.01, significant difference from corresponding air-exposed mice; ^###^ *P* < 0.001, significant difference from corresponding air-exposed mice. ^*^ *P* < 0.05, significant difference between CS-exposed WT mice and CS-exposed Ephx2^−/−^ mice.

Rn and Rrs of lung function were significantly increased after CS exposure in both WT and Ephx2^−/−^ groups (Fig. 2a, b). Also, Rn and Rrs indices in CS-Ephx2^−/−^ mice were lower than those of CS-WT mice (Fig. 2a, b). These data suggest that lungs of Ephx2^−/−^ mice are less susceptible to CS-induced airway resistance. However, Crs and Ers did not differ in the air-exposed and CS-exposed groups (Fig. 2c, d), perhaps due to insufficient CS exposure time to cause change in Crs and Ers.

### Ephx2^−/−^ Mice Show Attenuated Emphysema in Response to CS

**Fig. 3:**
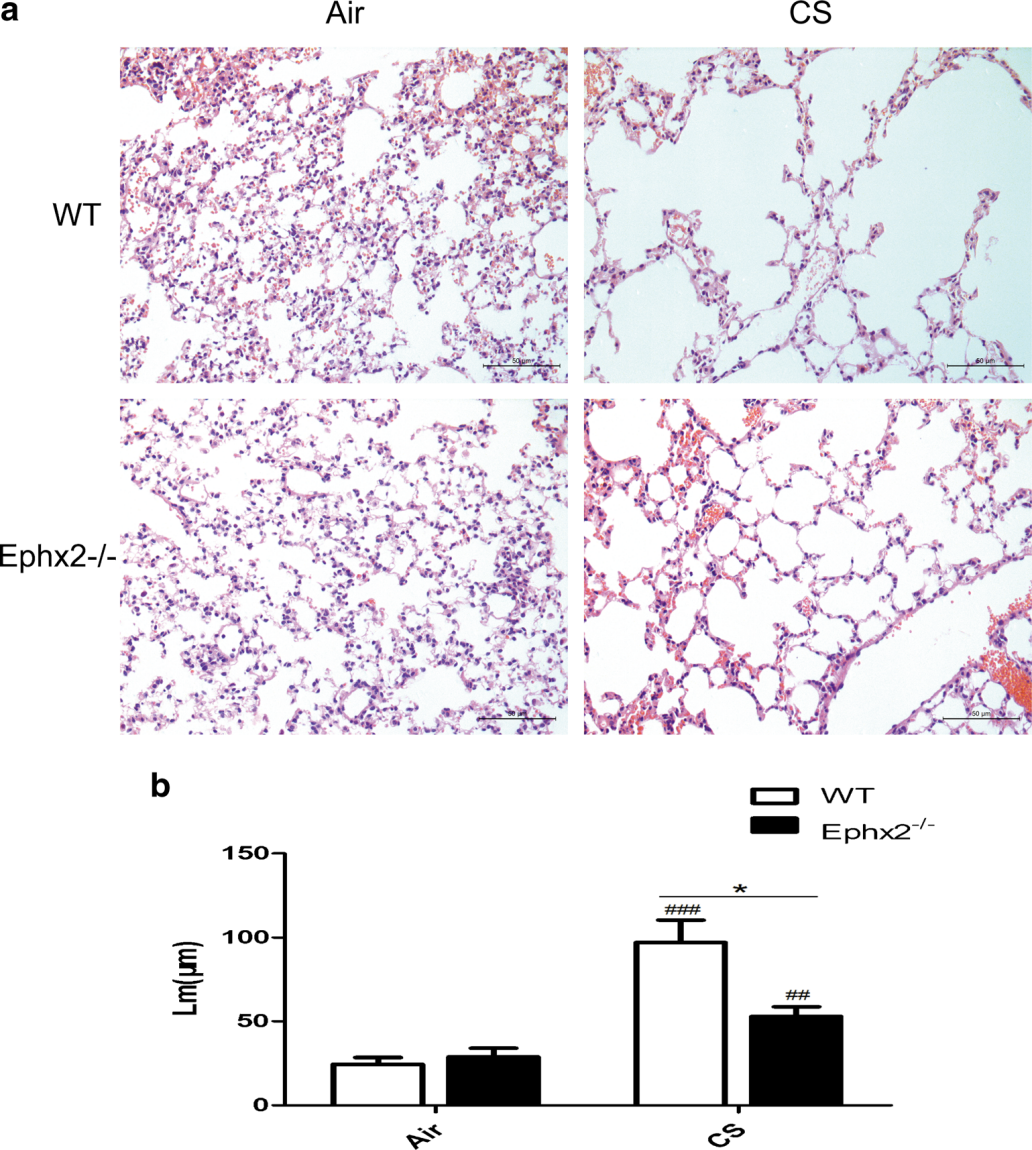
There is less air space enlargement in Ephx2^−/−^ mice in response to CS. **a** Representative H&E stained lung sections exposed to air and CS in WT and Ephx2^−/−^ mice. **b** Lm was calculated. Original magnification, ×100. Results are expressed as means ± SD (*n* = 3 mice/group). ^##^ *P* < 0.01, significant difference from corresponding air-exposed mice; ^###^ *P* < 0.001, significant difference from corresponding air-exposed mice. ^*^ *P* < 0.05, significant difference between CS-exposed WT mice and CS-exposed Ephx2^−/−^mice.

Pulmonary emphysema is a clinical phenotype of COPD, characterized by irreversible enlargement of airspaces distal to the terminal bronchiole and irreversible loss of alveolar structures [41]. Lm was measured to quantify emphysema and CS-exposed WT and CS-exposed Ephx2^−/−^ mice had greater alveolar size compared with corresponding air-exposed mice. Lm of CS-exposed Ephx2^−/−^ mice was significantly lower compared with CS-exposed WT mice (Fig. 3), indicating a partial protection against pulmonary emphysema.

### Ephx2^−/−^ Mice Have Less Inflammation in Response to CS

**Fig. 4:**
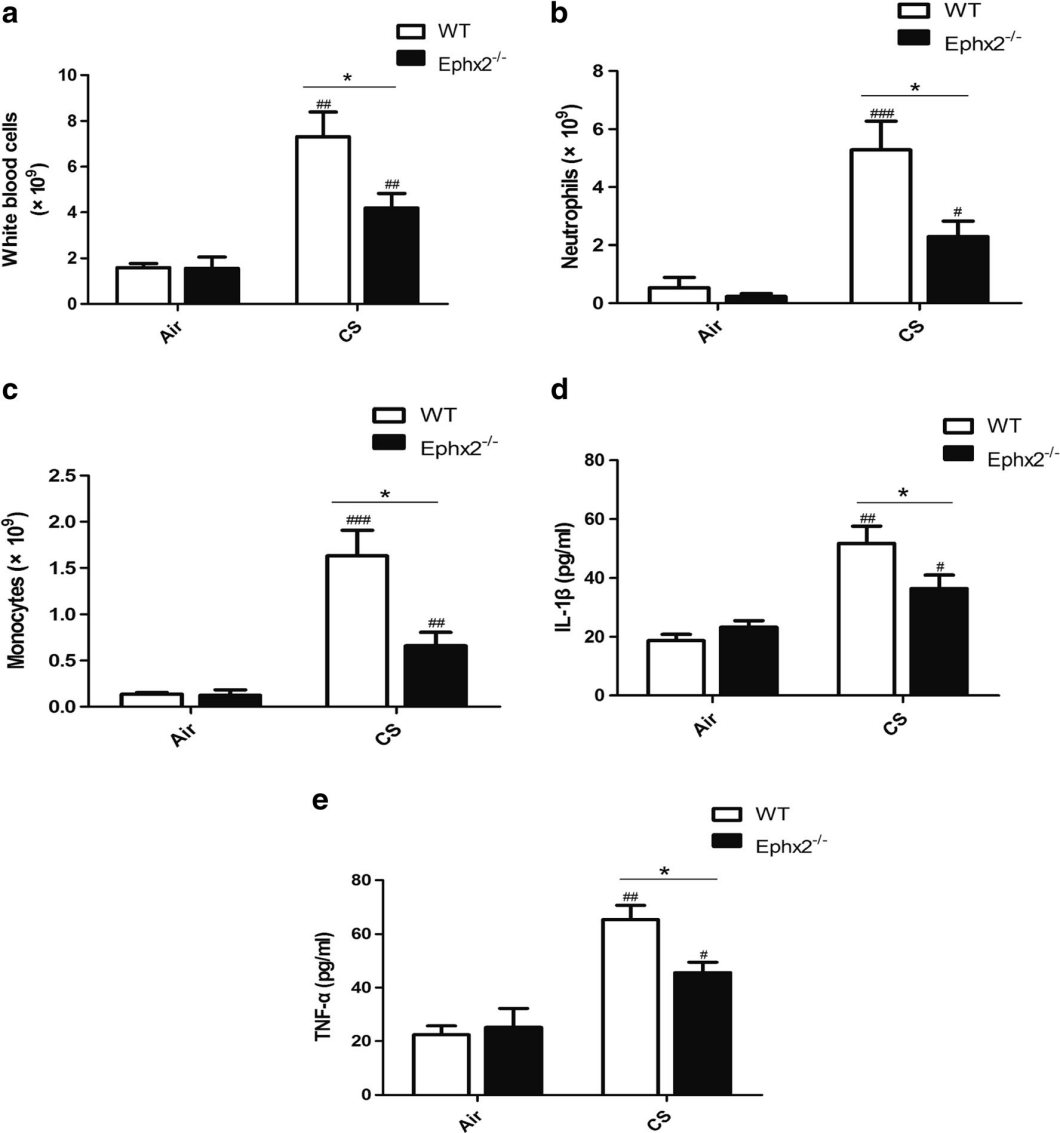
Ephx2^−/−^ mice had reduced inflammation in response to CS. After 12 weeks of exposure to air or CS, BALF were obtained and inflammatory cells and TNF-α and IL-1β were quantified. **a**–**c** CS-exposed WT mice and CS-exposed Ephx2^−/−^ mice had more leukocytes, neutrophils, and monocytes in BALF than air-exposed mice. CS-exposed Ephx2^−/−^ mice had significantly fewer inflammatory cells in BALF. **d**, **e** TNF-α and IL-1β were increased in CS-exposed WT and CS-exposed Ephx2^−/−^ mice than with air-exposed WT and air-exposed Ephx2^−/−^ mice. CS-exposed Ephx2^−/−^ mice had significantly lower TNF-α and IL-1β than CS-exposed WT mice. Results are expressed as means ± SD (*n* = 4 mice/group). ^#^ *P* < 0.05, significant difference from corresponding air-exposed mice; ^##^ *P* < 0.01, significant difference from corresponding air-exposed mice; ^###^ *P* < 0.001, significant difference from corresponding air-exposed mice; ^*^ *P* < 0.05, significant difference between CS-exposed WT mice and CS-exposed Ephx2^−/−^ mice.

Inflammatory factors, IL-1β, and TNF-α were measured in BALF and these factors were greater after 12 weeks of CS exposure both in WT and Ephx2^−/−^ mice compared with air-exposed mice (Fig. 4a–c). CS-exposed Ephx2^−/−^ mice had less inflammatory cell influx compared with CS-exposed WT mice and CS-exposed WT, and Ephx2^−/−^ mice had more TNF-α and IL-1β in BALF than the air-exposed WT and Ephx2^−/−^ mice (Fig. 4d, e). CS-exposed Ephx2^−/−^ mice had less TNF-α and IL-1β compared with CS-exposed WT mice (Fig. 4d, e). Thus, inflammation is aggravated after 12 weeks of CS exposure and Ephx2^−/−^ mice are protected against this to a degree.

### Ephx2^−/−^ Mice Have Reduced NF-κB Expression in Response to CS

**Fig. 5:**
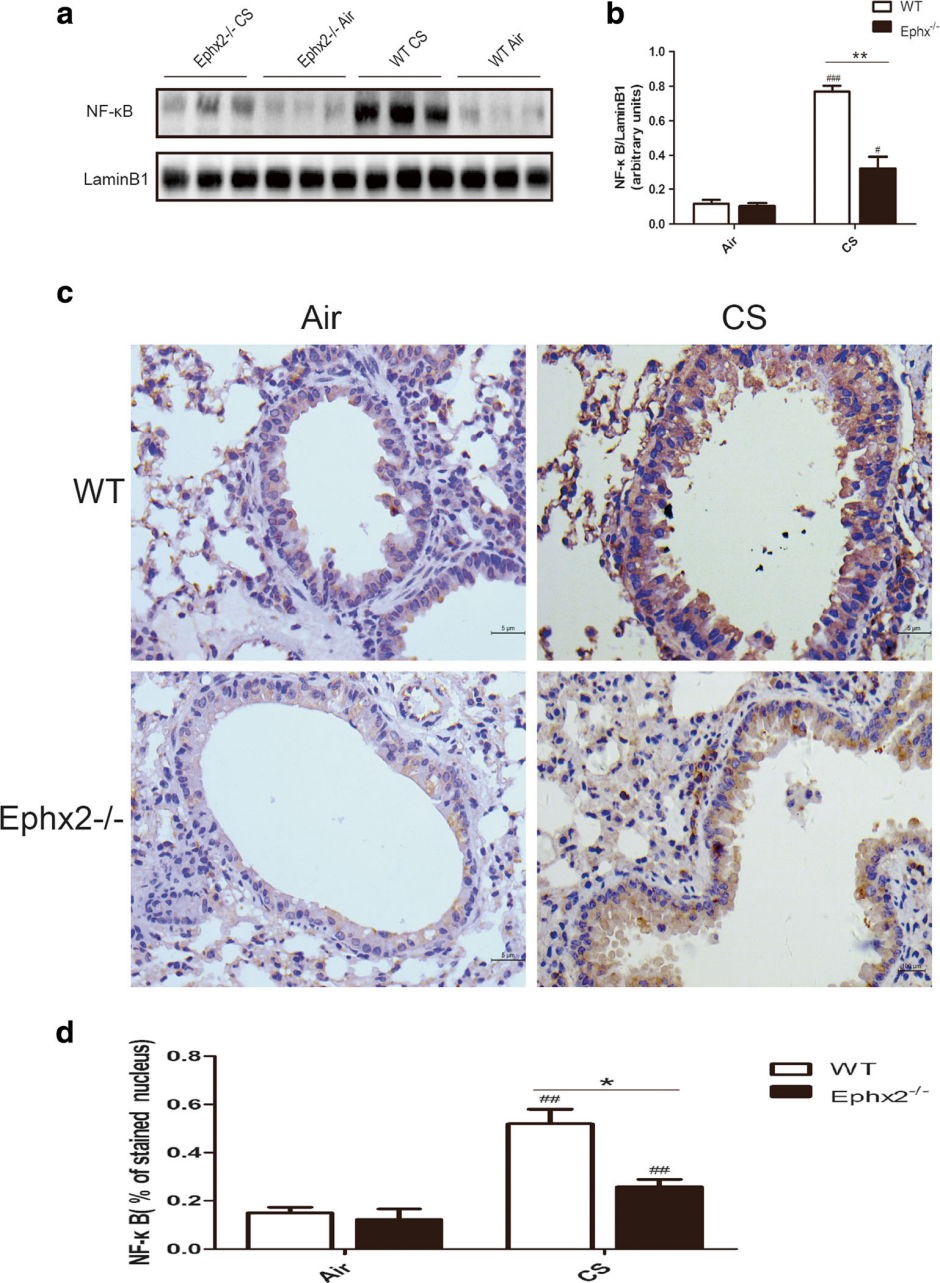
Nuclear factor NF-κB is expressed in the lungs in response to CS. NF-κB expression was measured after 12 weeks of exposure to air or CS. **a** Western blot for NF-κB expression in lung tissue. **b** Relative intensity of NF-κB calculated by densitometry. Results represent means ± SD from at least three independent experiments. **c** NF-κB expression in lung alveolar/airway epithelial cells from each group via immunohistochemistry (*n* = 3 mice/group). Inverted phase contrast microscopy revealing dark brown staining representing NF-κB expression (×400). **d** Graph is IntDen of NF-κB. ^#^ *P* < 0.05, significant difference from corresponding air-exposed mice; ^##^ *P* < 0.01, significant difference from corresponding air-exposed mice; ^###^ *P* < 0.001, significant difference from corresponding air-exposed mice; ^*^ *P* < 0.05, significant difference between CS-exposed WT mice and CS-exposed Ephx2^−/−^ mice; ^**^ *P* < 0.01, significant difference between CS-exposed WT mice and CS-exposed Ephx2^−/−^ mice.

NF-κB was measured and was observed to be greater in CS-exposed WT mice and CS-exposed Ephx2^−/−^ mice compared to air-exposed mice (Fig. 5a, b). Ephx2^−/−^ mice had reduced NF-κB expression compared to WT mice in response to CS (Fig. 5a, b). Immunohistochemical data were consistent with blot data (Fig. 5c, d) and blot and immunohistochemistry results were consistent with findings of greater inflammatory factors and cells in BALF (Fig. 4). Thus, Ephx2 deficiency may increase resistance to inflammation via the NF-κB pathway.

### Ephx2^−/−^ Mice Have Reduced Autophagy in Response to CS

**Fig. 6:**
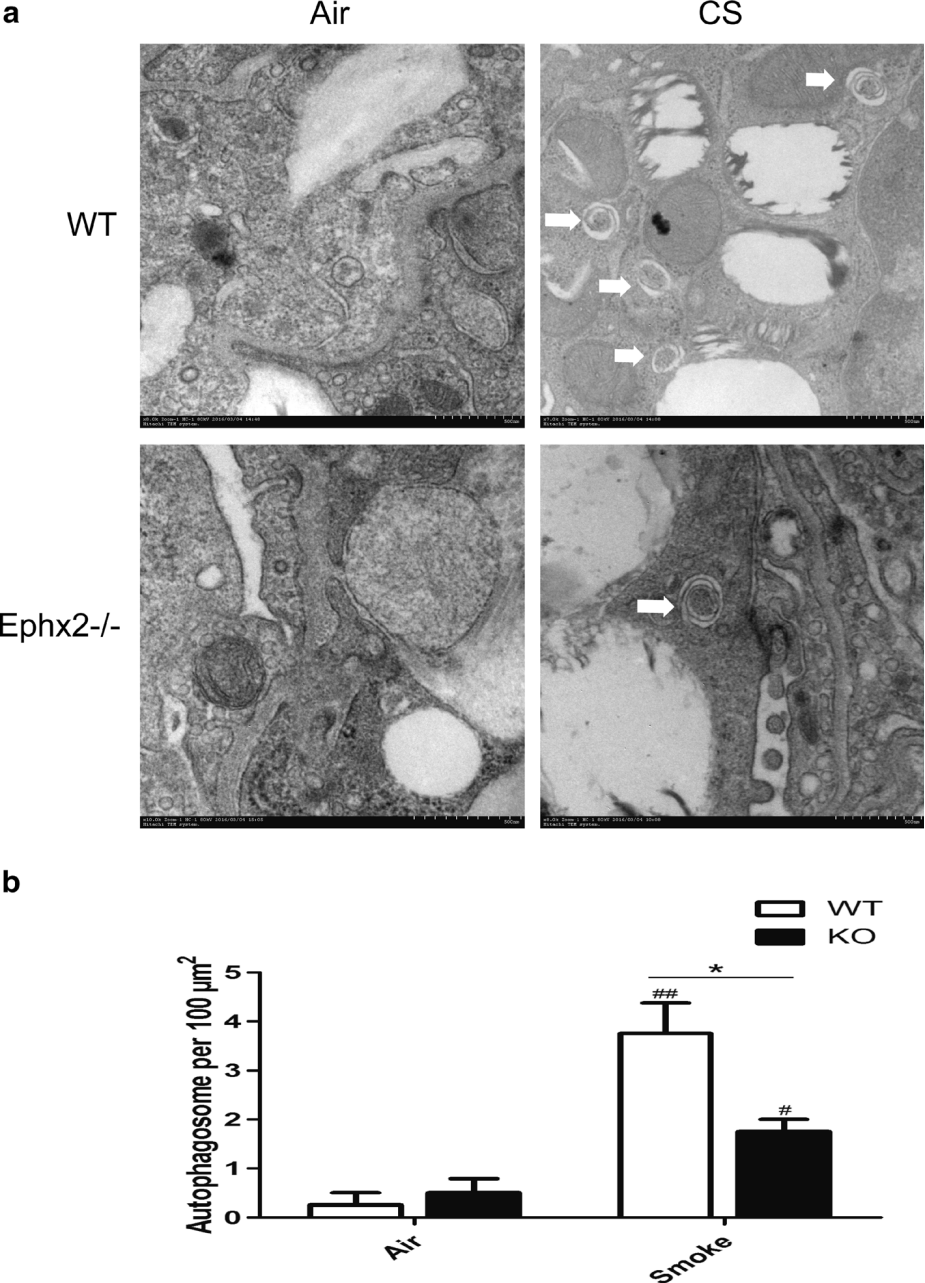

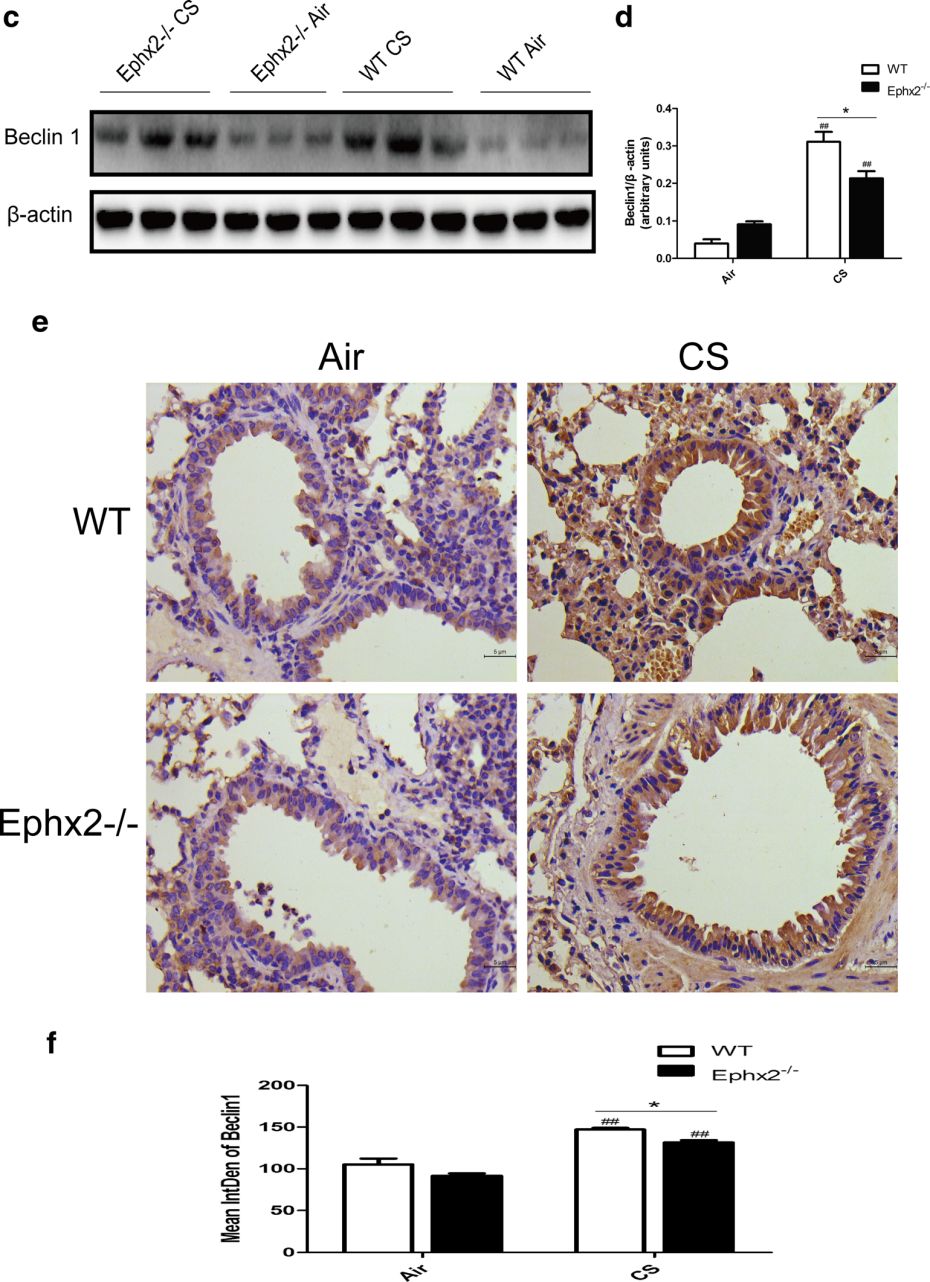

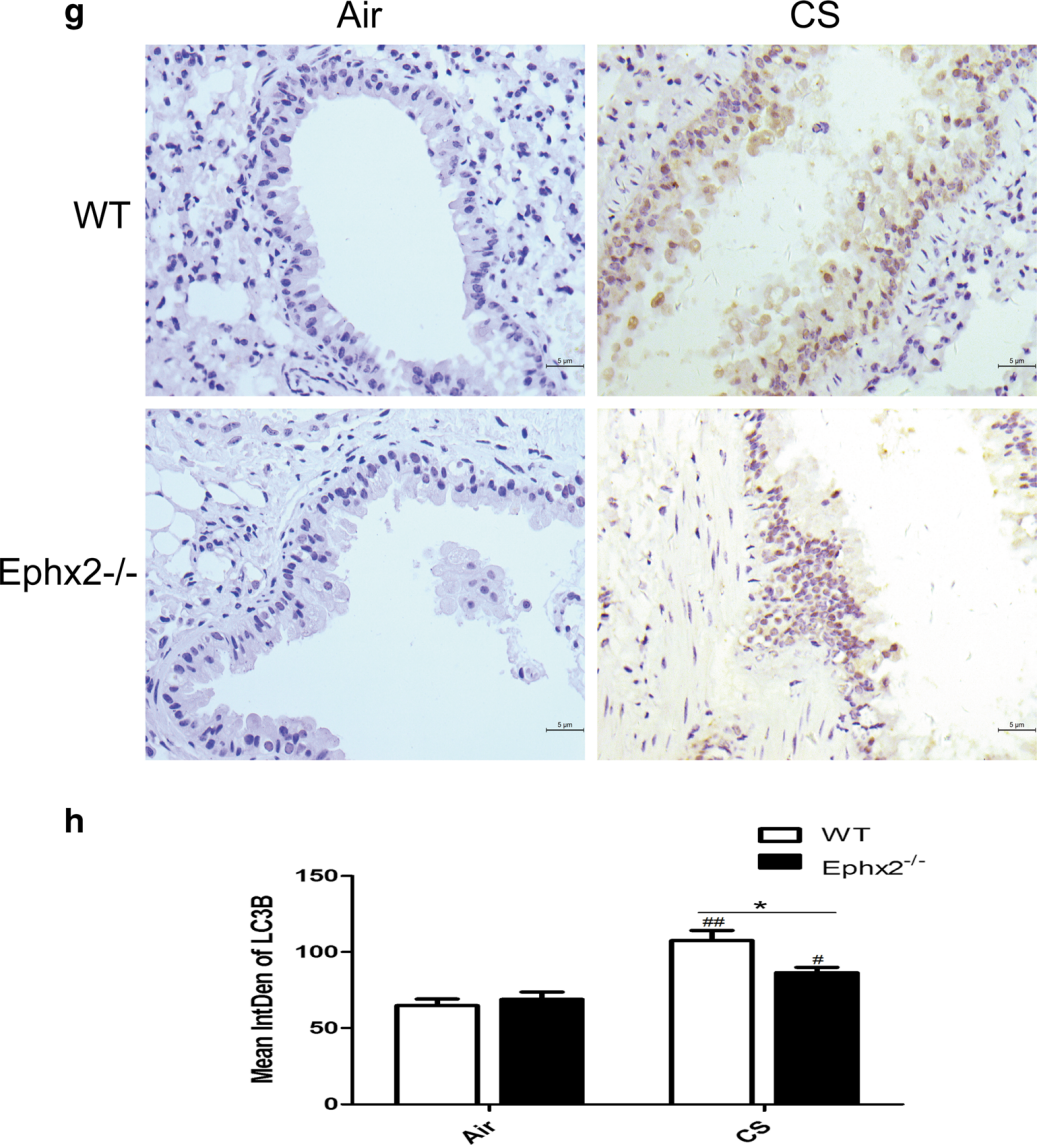
Ephx2^−/−^ mice had less autophagy in response to CS-WT and Ephx2^−/−^ mice were exposed to air or CS for 12 weeks and autophagy protein LC3B and Beclin 1 expression was measured. **a** Autophagosomes under electron microscopy (×100,000); *white arrows* depict autophagosomes (*n* = 3 mice/group). **b** EM images scored autophagosomes. Data are expressed as AVs/100 μm^2^ (*N* = 10 images representative of each group). **c** Western blot of Beclin 1. **d** Relative intensity of Beclin 1 was calculated by densitometry and values are presented as means ± SD. **e**–**h** Representative immunohistochemical images for Beclin 1 (**e**) and LC3B (**g**) in mouse lung tissues (*n* = 3 mice/group). **f**, **h** Graph represents IntDen of Beclin 1 and LC3B and values are presented as means ± SD. ^#^ *P* < 0.05, significant difference from corresponding air-exposed mice; ^##^ *P* < 0.01, significant difference from corresponding air-exposed mice. ^*^ *P* < 0.05, significant difference between CS-exposed WT mice and CS-exposed Ephx2^−/−^ mice.

Autophagy contributes to COPD [3] and may interact with EETs [19, 35]. For this reason, autophagy was measured in each mouse group (Fig. 6a). Data show that autophagosomes increased in CS-exposed WT and CS-exposed Ephx2^−/−^ mice compared to air-exposed animals. CS-exposed Ephx2^−/−^ mice had fewer autophagosomes than CS-exposed WT mice (Fig. 6a, b). In this way, CS can induce autophagy in a COPD mouse model and Ephx2^−/−^ mice may be less susceptible to autophagy. Data also show that Beclin 1 and LC3B (Fig. 6c, e, g) were significantly higher in CS-exposed WT and CS-exposed Ephx2^−/−^ mice than in air-exposed mice and that CS-exposed Ephx2^−/−^ mice had less Beclin 1 and LC3B expression compared to CS-exposed WT.

## DISCUSSION

COPD is mainly caused by cigarette smoking, and it is characterized by emphysema, pulmonary inflammation, fibrogenesis, and reduced lung function [31]. Increased autophagy is also observed in lungs of mice exposed to chronic CS [3]. The greatest amount of airway inflammation was observed in mice after 12 weeks of CS exposure, and WT and Ephx2^−/−^ mice both had aggravated emphysema, increased airway resistance, and inflammation and autophagy compared to air-exposed animals, but Ephx2^−/−^ mice had fewer of these symptoms than WT mice and they had reduced autophagy. In this way, Ephx2 deficiency may protect individuals from CS-induced lung disease.

CYP-mediated arachidonic acid metabolism is an important regulator of inflammation and EETs, whose CYP epoxygenase-derived products are proposed to be anti-inflammatory mediators in vascular, liver, and renal diseases [29]. EETs are hydrolyzed by sEH, which is encoded by Ephx2^−/−^ gene [28], to DHETs, which has a less important biological function. Inhibitors targeting sEH-induced increasing endogenous EETs can be antihypertensive and anti-inflammatory as well as protective to the brain, heart, and kidneys [11, 24]. Recently, EETs and sEH inhibitors have been shown to have an anti-inflammatory effect in lung disease. Morin’s group reported that 14, 15-EET has anti-inflammatory effects on human bronchi treated with TNF-α [23]. Ma and colleagues reported that 11, 12-EET and 14, 15-EET can reduce CS extraction-induced release of IL-8 in human bronchial epithelial cells [20]. Podolin’s group demonstrated that a sEH inhibitor reduces CS-induced pulmonary inflammation by inhibiting its initiation and promoting its resolution [32]. Recently, an initial report indicated that inhibition of sEH downregulates the inflammatory response induced by acute or sub-chronic tobacco smoke exposure, which was consistent with data from Ephx2^−/−^ knockout mice [30]. This was the first report to describe chronic CS-induced inflammatory response in an Ephx2^−/−^ knockout mice model. Current data showed that 12 weeks of CS exposure elevated inflammatory cells in BALF of WT and Ephx2^−/−^ mice but that, Ephx2^−/−^ mice had less inflammatory cell influx than WT mice. TNF-α and IL-1β displayed the same trend. NF-κB expression was noted to be elevated in CS-exposed WT and Ephx2^−/−^ mice, and Ephx2^−/−^ mice had less NF-κB than WT mice. Immunohistochemistry and blot data were consistent with these findings.

EETs are reported to inhibit the NF-κB signaling pathway, and subsequently inhibit expression of vascular cell adhesion molecule-1 (VCAM-1), attenuating leukocyte migration [29]. Nording’s group reported that 8, 9-EET/DHET and 14, 15-EET/DHET ratios in plasma were more elevated in Ephx2^−/−^ mice subjected to after acute and sub-chronic CS exposure than in WT mice, suggesting an anti-inflammatory property of Ephx2 deficiency [30]. In this way, Ephx2 deficiency may lead to increased EETs/DHET ratios which may block the NF-κB signaling pathway and protect against chronic CS-induced lung inflammation. The manner by which this occurs is uncertain.

In this way, Ephx2^−/−^ mice are less susceptible to CS-induced emphysema and airway resistance and air space enlargement is greater in WT and Ephx2^−/−^ mice in response to CS exposure but comparatively less in CS-exposed Ephx2^−/−^ mice. Pulmonary emphysema occurs in COPD and is characterized by irreversible enlargement of airspaces distal to the terminal bronchiole and irreversible loss of alveolar structures [41]. Previous reports have suggested that infiltration of neutrophils and macrophages with release of proteolytic enzymes in response to CS contributes to lung tissue destruction in mouse emphysema models [7, 10, 38]. Apoptosis triggered by oxidative stress can also contribute to pathogenesis of CS-induced emphysema [14].

Ephx2^−/−^ mice have reduced inflammation and EETs have a role in an anti-apoptotic CS-induced human bronchial epithelial cell line. In this way, the mechanism of repair of tissue in Ephx2^−/−^ mice with emphysema may depend on EET properties. COPD features elevation of respiratory resistance and reduced compliance [2, 43]. Yang’s group reported that respiratory system resistance indices were elevated after 10 or 12 months of tobacco inhalation [45]. This is consistent with the current observations. After 12 weeks of CS exposure, Rn and Rrs increased in both exposed animal groups even though Ephx2^−/−^ mice had normal Rn and Rrs levels and WT mice did not. This may be partly due to the lower concentrations of fewer inflammatory cells and inflammatory factors. There was no significant difference between the CS-exposed group and the air-exposed group with respect to Crs or Ers likely because 12 weeks is not sufficient for these to change appreciably. Ephx2^−/−^ mice gained less weight than WT but why this is so is unclear.

Ephx2 deficiency inhibits autophagy in a CS-exposed COPD mouse model. Data showed there to be more autophagosomes in lung tissues of CS-exposed WT mice and CS-exposed Ephx2^−/−^ mice than in animals exposed to air, and CS-exposed Ephx2^−/−^ mice had fewer autophagosomes than WT mice. In this way, autophagy was enhanced in a COPD mouse model and Ephx2 deficiency inhibited autophagy. Beclin 1 and LC3B data are consistent with these trends. Chen’s group reported for the first time that increased autophagy is associated with CS-induced COPD [3], and autophagy was induced in the current COPD model. An and colleagues reported that TLR4 exerts a protective role in CS-induced emphysema development by reducing the autophagic pathway [1]. Ji’s group reported that Vam3 decreases CS-induced autophagy and inhibits oxidative stress. Stefan and colleagues proposed that autophagy has an effect on the stress response to CS exposure *in vitro* and may contribute to the etiology of COPD [34]. Two reports have described EETs and heart and liver protection via autophagy regulation [19, 35]. In this way, Ephx2^−/−^ mice may have protection against CS-induced lung inflammation and this likely takes place through reduced autophagy but EETs and autophagy interactions require elucidation to confirm these assertions. In addition to the protective role of autophagy suppression we proved, autophagy is a double-edge sword and it has a complex cross-talk with apoptosis [33]. Autophagy primarily plays a protective role that may prevent cell death and it has been demonstrated that autophagy plays a critical role in maintaining cellular homeostasis and the adaption to environmental stress (oxidative stress, starvation, hypoxia, *etc*.) [13–15, 22]. However, it has been proved that both excessive and impaired autophagy can cause cell death in some model systems due to the interaction with apoptosis [33]. Deficiency of LC3B and Beclin 1 inhibits cell death induced by CSE in pulmonary epithelial cells and the airspace enlargement and apoptosis after cigarette smoke exposure are inhibited in LC3B-deficient mice [3, 4, 14]. Further studies are required to elucidate the role of autophagy in COPD. In conclusion, we used mouse model with a deficiency in Ephx2 to explore the effect of this gene on chronic CS-induced COPD. Mice lacking this gene had less lung inflammation and reduced emphysema and airway resistance. Also, the gene may contribute to less autophagy. In this way, this may represent a novel therapeutic target for the treatment of COPD.
